# Investigation of Blood Count-Based Inflammatory Biomarkers as Predictors of Response to Dupilumab Treatment in Patients with Chronic Rhinosinusitis with Nasal Polyps

**DOI:** 10.3390/pharmaceutics16111370

**Published:** 2024-10-25

**Authors:** Michael Habenbacher, Ulrich Moser, Ahmed Abaira, Peter Kiss, Clemens Holzmeister, Jakob Pock, Katharina Walla, Angelika Lang, Alexandros Andrianakis

**Affiliations:** Department of Otorhinolaryngology, Medical University of Graz, Auenbruggerplatz 26, 8010 Graz, Austria

**Keywords:** biologics, dupilumab, chronic rhinosinusitis, CRSwNP, biomarker, otorhinolaryngology

## Abstract

Background/Objectives: Chronic rhinosinusitis with nasal polyps (CRSwNP) is a type 2 inflammatory disease often resistant to standard treatments. Dupilumab, a monoclonal antibody targeting the IL-4α receptor, has shown efficacy in CRSwNP, but a significant subset of patients do not respond to this therapy. This study aims to investigate pretreatment complete blood count (CBC)-based inflammatory biomarkers as predictors of response to dupilumab in patients with CRSwNP. Methods: This mono-centric, retrospective, single-arm longitudinal cohort study included 80 patients with uncontrolled CRSwNP who received dupilumab treatment at the Medical University of Graz. Patients were classified into responder and non-responder groups based on a reduction of >1 in nasal polyp score (NPS) and a sinonasal outcome test-22 (SNOT-22) score <40 points at six months. Pretreatment CBC-derived biomarkers, including eosinophil count, neutrophil-to-lymphocyte ratio (NLR), platelet-to-lymphocyte ratio (PLR), and systemic inflammation indices including the aggregate inflammation systemic index (AISI), systemic inflammation index (SII), and systemic inflammation response index (SIRI), were analyzed for their predictive value. Results: Of the 80 patients, 72.5% were classified as responders, while 27.5% were non-responders. A significant positive correlation was found between baseline eosinophil count and NPS reduction (*p* = 0.027), suggesting that higher eosinophil levels may predict higher NPS reduction in dupilumab treatment. However, no significant associations were observed between NLR, PLR, and systemic inflammation indices with treatment outcomes. Conclusions: Pretreatment eosinophil count may serve as a potential biomarker for predicting nasal polyp reduction in dupilumab treatment of CRSwNP. Other CBC-based inflammatory markers did not show significant predictive value. Further prospective studies are needed to validate these findings and explore additional, reliable biomarkers to optimize treatment outcomes for CRSwNP patients.

## 1. Introduction

Chronic rhinosinusitis (CRS) is a common health condition, affecting approximately 5–12% of people globally, and stands as one of the most frequent chronic diseases worldwide. CRS is a chronic inflammation of the nose and paranasal sinuses defined as persistence of two or more typical sinonasal symptoms (nasal blockage/congestion, impaired sense of smell, rhinorrhea, post-nasal drip, facial pain/pressure) lasting for more than 12 weeks, together with objective inflammatory signs in nasal endoscopy and/or radiological imaging. Based on the presence of nasal polyps, CRS is traditionally categorized into those with nasal polyps (CRSwNP) and those without (CRSsNP), with CRSwNP being the more severe manifestation of the disease [1,2]. CRSwNP represents around one-third of CRS cases, with a prevalence estimated between 2% and 4% in the Western world [1,3,4,5]. CRSwNP is frequently linked with coexisting conditions including asthma bronchiale and NSAID-exacerbated respiratory disease (N-ERD) [1,6,7,8].

The precise cause of CRS remains unclear, but it is believed to involve several factors, including an abnormal host response to irritants, commensal and harmful microorganisms, as well as allergens. Additionally, it may be linked to obstruction of sinus drainage pathways, dysfunction of the mucociliary clearance system, or underlying infections. The hallmark of CRS pathophysiology is the disruption of the protective barrier in the mucosa of the paranasal sinuses, leading to a persistent and unresolved inflammatory response. Recent progress in the comprehension of CRS pathophysiology has resulted in the creation of an endotype-based classification framework, dividing CRS into three different endotypes (type 1–3) based on the underlying molecular pathways of mucosal inflammation [1]. Approximately 85% of CRSwNP cases in the Western world fall under the type 2 category, which is also the most extensively studied [9,10]. Conversely, type 1 inflammation is more prevalent among Asian people [11]. The type 2 inflammatory process is marked by the infiltration and accumulation of eosinophils and mast cells within the affected mucosal tissue. This cascade starts when alarmins such as TSLP trigger group 2 innate lymphoid cells (ILC2) and induce dendritic cells, which subsequently drive the expansion of T helper 2 (Th2) lymphocytes. Once activated, ILC2 and Th2 cells produce significant amounts of interleukin-4 (IL-4), IL-5, and IL-13, which are recognized as the central drivers of type 2 inflammation. IL-4 serves as a key differentiation factor, guiding naive CD4+ T cells toward the Th2 phenotype. Additionally, it functions as a growth factor for B cells, triggers the isotype switch to immunoglobulin E (IgE), and promotes eosinophil activation and chemotaxis. IL-13 also plays a significant role in isotype switching and eosinophil (EOS) chemotaxis, while being primarily responsible for goblet cell hyperplasia, mucus production, airway hyperresponsiveness, and smooth muscle contraction. The signaling pathways of IL-4 and IL-13 operate through two possible heterodimeric receptors that share a common receptor component, known as the IL-4α chain [1,12,13,14].

Standard treatment of CRSwNP involves the long-term use of topical corticosteroids (COS), short-term courses of systemic COS, and endoscopic sinus surgery (ESS) [1]. Nevertheless, even with these standard treatments, a significant proportion of CRSwNP patients continue to suffer from ongoing symptoms or experience recurrence disease [15,16,17]. Recent therapeutic advancements in uncontrolled CRSwNP have introduced biologic medications. Biologicals are medicinal products that are generated through biological processes. One class of biologics are monoclonal antibodies (mAb), produced using recombinant DNA technology. mAb target a specific epitope in the body, such as inflammatory mediators or immune cells involved in the pathophysiological pathways responsible for chronic inflammatory conditions [18]. Several mAb have been approved for the treatment of type 2 immune-mediated diseases including atopic dermatitis [19], asthma [20], eosinophilic esophagitis [21], food allergies [22] and CRSwNP [23]. At present, three mAb have been authorized as add-on treatments for managing uncontrolled CRSwNP, namely dupilumab in 2019 [24], omalizumab in 2020 [25], and mepolizumab in 2021 [26]. Dupilumab, a fully human mAb, specifically binds to the α-subunit of the IL-4 receptor. Through doing so, dupilumab blocks the signaling pathways of both IL-4 and IL-13, thereby effectively reducing type 2 inflammation. The efficacy of dupilumab treatment in CRSwNP has been confirmed in randomized clinical trials and in several real-life studies [27]. However, approximately 40% of the patients do not respond well to biologic treatment [28,29].

Various clinical and biological biomarkers have been studied as potential predictors of response to dupilumab therapy in CRSwNP, yet consistent evidence supporting their reliability remains lacking [30,31]. Therefore, there is a need for reliable and easily obtainable predictive biomarkers for response to dupilumab therapy in CRSwNP. Recent studies have highlighted the increasing interest in blood cell count-derived inflammation indices as potential predictors of therapeutic outcomes in various inflammatory conditions [32,33,34,35,36]. These indices, such as the neutrophil-to-lymphocyte ratio (NLR) and platelet-to-lymphocyte ratio (PLR), may provide easy-accessible and cost-effective markers of systemic inflammation. In the context of CRSwNP, several studies have investigated the prognostic value of these inflammatory biomarkers [37,38]. According to a recent meta-analysis, elevated preoperative NLR are associated with an increased risk of nasal polyp recurrence after ESS [37]. More recently, researchers had suggested that a higher PLR may correlate with a more favorable response to dupilumab treatment in CRSwNP patients. However, the authors stressed that further data are needed to confirm these results [39]. Additionally, another study observed a similar positive association between elevated PLR and improved dupilumab efficacy in patients with AD, another type 2 inflammatory disease. This study also reported that dupilumab response was linked to other blood-based inflammatory markers, including the aggregate inflammation systemic index (AISI), systemic inflammation index (SII), and systemic inflammation response index (SIRI) [40]. To our knowledge, no prior studies have evaluated the prognostic value of these biomarkers in CRSwNP patients undergoing dupilumab therapy.

This study aimed to explore the association between pretreatment CBC-based inflammatory biomarkers and the therapeutic response to dupilumab in patients with CRSwNP. Through identifying predictive biomarkers, this research sought to enhance the precision of treatment approaches for this chronic and burdensome condition.

## 2. Materials and Methods

### 2.1. Study Design

The present study was a mono-centric, retrospective, single-arm, longitudinal cohort analysis conducted at the Department of Otorhinolaryngology at the Medical University of Graz, Austria. The study was conducted in accordance with the Declaration of Helsinki and approved by the Institutional Review Board of the Medical University of Graz (1086/2024).

### 2.2. Study Population and Eligibility Criteria

All patients with primary, uncontrolled CRSwNP, who received dupilumab treatment 300 mg biweekly (DUPIXENT^®^, SANOFI WINTHROP INDUSTRIE, 82 Avenue Raspail 94250 Gentilly France) between April 2020 and March 2024, were included in this study. The diagnosis of primary CRSwNP was established according to the criteria outlined in the European Position Paper on Rhinosinusitis and Nasal Polyps (EPOS) [1]. Patients were excluded in case of secondary CRSwNP, incomplete medical records, if they failed to attend the 6-month follow-up, or if they discontinued dupilumab treatment before the 6-month follow-up.

### 2.3. Indication for Dupilumab Therapy

Dupilumab was prescribed to CRSwNP patients based on the following modified EPOS indication criteria for biologics [1]: (1) the presence of nasal polyps, (2) a history of ESS or a medical contraindication to surgery, and (3) the fulfillment of at least three of the following five conditions: I. evidence of type 2 inflammation (defined as a blood eosinophil count of ≥0.25 × 10^9^/L or total serum IgE levels of ≥100 kU/L); II. the requirement for two or more courses of systemic COS annually; III. a sinonasal outcome test-22 (SNOT-22) score of ≥40; IV. a reduced sense of smell (as indicated by a score of ≥1 on the “smelling” subitem of the SNOT-22 or by hyposmia on the sniffing sticks identification screening test 12 [SSIT-12]. This definition was modified from the EPOS criteria as smell testing is not performed routinely at our clinic); or V. comorbid asthma necessitating regular inhaled medications, or a history of N-ERD.

### 2.4. Clinical Protocol

At the initial consultation, patients underwent a clinical examination that included nasal endoscopy to determine nasal polyp size. Their medical history—including age, sex, previous ESS, and comorbidities such as asthma and NERD—was also reviewed, and a blood sample was collected to assess CBC (leukocytes, platelets, neutrophils, monocytes, lymphocytes, eosinophils) and IgE. Once the indication for biologic therapy was confirmed, the first dose of 300 mg dupilumab s.c. was administered at our outpatient clinic, approximately 7 to 14 days later, under medical supervision. Dupilumab was subsequently applied every two weeks. The outcome of dupilumab treatment was then assessed during a follow-up visit six months later.

### 2.5. Outcome Parameters

The primary outcome parameter of this study was the response to dupilumab treatment. The Austrian health care system defines response to dupilumab as a reduction of >1 point in the nasal polyp score (NPS) [41]. In our department, in addition to this objective criterion, a SNOT-22 score of <40 points was used as a patient-reported response criterion, as this cut-off value corresponds to symptom control [1,42]. Patients were categorized as either responders or non-responders based on these two criteria. We evaluated various pretreatment CBC-derived inflammatory biomarkers, including EOS, NLR, PLR, SIRI, SII, and AISI, for their potential prognostic impact. We used the Meltzer NPS, which rates nasal polyps from 0 to 4 on each side, for a total score of 0 to 8. A score of 0 means no polyps, 1 indicates polyps in the middle meatus, 2 means multiple polyps filling the middle meatus, 3 indicates polyps extending beyond the middle meatus, and 4 means the nasal cavity is fully blocked with polyps [43]. The SNOT-22 is a CRS-specific quality of life questionnaire. It consists of 22 questions covering symptoms such as nasal issues, ear and facial pain, psychological effects, and sleep problems. Patients score their symptoms from 0 to 5, with a higher total score (up to 110) indicating more severe symptoms [1,44].

### 2.6. Statistics

Statistical analysis was conducted using SPSS © software, version 29.0 (IBM©, Armonk, NY, USA). Continuous variables were reported as mean ± standard deviation or median (range) as appropriate, while categorical variables were presented as absolute numbers and percentages. Independent *t*-tests were used to compare continuous variables between groups, while Pearson’s chi-squared test was applied for categorical variables. Pearson’s correlation analyses between biomarkers and treatment outcomes were performed. A univariate and multivariate binary logistic regression analysis, with an “enter method” and analyzed with confounders (age, sex, number of previous ESS, coexisting asthma, coexisting NERD), was utilized to evaluate the independent prognostic impact of the biomarkers on response. Statistical significance was set at *p* < 0.05.

## 3. Results

### 3.1. Demographics and Clinical Baseline Characteristics

This study involved 80 individuals, comprising 36 women (45%) and 44 men (55%), diagnosed with CRSwNP who received dupilumab therapy and fulfilled eligibility criteria. Patients showed an average age of 50.8 ± 14.6 years at the time of treatment initiation. The total cohort had a median count of previous ESS of 2, with a range from 0 to 7. For medical issues, two patients (2.5%) were unable to go through any prior ESS procedures. A total of 19% (n = 15) of the patients met three of the five indication criteria, 45% (n = 36) met four criteria, and 36% (n = 29) fulfilled all five criteria. The most frequently observed criteria were an evidence of type 2 inflammation and an impaired sense of smell, affecting 95% (n = 76) of the individuals. A baseline SNOT-22 score ≥40 was present in 78% (n = 62), while 76% (n = 61) required two or more courses of systemic corticosteroids annually. Additionally, 67% (n = 54) had coexisting asthma. Furthermore, 19% (n = 15) of the patients were diagnosed with N-ERD. The mean value of leukocytes, platelets, neutrophils, monocytes and lymphocytes were 7.2 ± 1.6 × 10^9^/L, 259 ± 53.4 × 10^9^/L, 4.2 ± 1.3 × 10^9^/L, 0.6 ± 0.6 × 10^9^/L and 1.9 ± 0.4 ×10^9^/L, respectively.

Out of the total cohort, 72.5% (n = 58) of the patients showed a good treatment response (=responders group), while the remaining 27.5% (n = 22) did not response well to the treatment (=non-responders group). Table 1 presents a detailed overview of demographic and clinical characteristics, categorized by response groups. Statistical analysis revealed no significant differences in baseline characteristics between the groups (*p* > 0.05). The response group showed a significant higher total reduction in NPS [t(78) = 4.3, *p* < 0.001, d = 1.1], in SNOT-22 [t(78) = 4, *p* < 0.001, d = 1] and in sense of smell SNOT-22 subitem [t(78) = 4, *p* < 0.001, d = 1].

### 3.2. Total Cohort Treatment Outcome

In the entire cohort, there was a statistically significant reduction in NPS, which dropped by an average of 3.7 ± 2.1, from a baseline value of 4.8 ± 1.7 to 1.2 ±1.4 at follow-up [t(79) = 15.7, *p* < 0.001, d = 1.7]. At the follow-up visit, 69 patients (86%) had an NPS reduction of >1. The SNOT-22 score also showed significant improvement, decreasing by an average of 30.4 ± 21.9, from an initial value of 55.5 ± 21 to 25.1 ± 19.3 [t(79) = 12.4, *p* < 0.001, d = 1.4]. In total, 63 patients (78%) achieved a SNOT-22 score <40.

### 3.3. Comparison of CBC-Based Inflammatory Biomarkers Between Response-Groups

For the total cohort, the mean EOS, IgE, NLR, PLR and LMR were 0.5 ± 0.9 × 10^9^/L, 199.3 ± 234.3 kU/L, 2.3 ± 1.1, 141.4 ± 37.1, and 3.4 ± 1.2, respectively. Furthermore, the mean SIRI, SII and AISI were 1.5 ± 1.5, 601.1 ± 285, and 405.2 ± 475, respectively. Regarding response groups comparison, there were no statistically significant differences (*p* > 0.05). Detailed results of response group comparisons are displayed in Table 2.

### 3.4. Correlation Analyses

We further performed correlation analyses between CBC-based inflammatory biomarkers and treatment outcome parameters (total change in NPS and total change in SNOT-22), in order to evaluate if higher biomarker values are associated with a better treatment outcome.

#### 3.4.1. Total Change in NPS

A significant positive correlation was found between baseline EOS count and total change in NPS (ρ = 0.248; *p* = 0.027), indicating that higher baseline EOS are associated with a higher reduction of NPS. There were no significant correlations between total change in NPS and the remaining inflammatory biomarkers (*p* > 0.05). Detailed results are depicted in Table 3.

#### 3.4.2. Total Change in SNOT-22

No statistically significant correlations were found between pretreatment CBC-based inflammatory markers and total change in SNOT-22. Comprehensive data of the analyses are provided in Table 4.

### 3.5. Binary Logistic Regression Analysis

To independently evaluate the ability of CBC-derived inflammatory markers to forecast response to dupilumab treatment, we performed a binary logistic regression analysis. However, both univariate and multivariate analyses failed to yield statistically significant outcomes (*p* > 0.05). All model coefficients, odds and *p*-values can be found in Table 5.

## 4. Discussion

This study aimed to evaluate the prognostic value of pretreatment CBC-based inflammatory markers in predicting the clinical response to dupilumab in patients with CRSwNP. Our key findings revealed that baseline eosinophil count was significantly correlated with a reduction in NPS, suggesting that eosinophil levels may serve as a predictive biomarker for dupilumab effectiveness. However, other markers such as NLR, PLR, and systemic inflammation indices did not show significant associations with treatment outcomes.

Dupilumab has significantly enhanced the quality of life of individuals dealing with uncontrolled CRSwNP. A range of real-world studies have supported the drug’s effectiveness in managing these cases [27]. The outcomes seen in our cohort correspond closely with those observed in previous clinical trials [24] and real-life studies [27], particularly in terms of both clinical measures such as NPS and patient-reported outcomes such as SNOT-22 after six months of treatment. This consistency suggests that our cohort is representative of the broader patient population that typically responds well to dupilumab therapy, as noted in the literature.

Both ESS and dupilumab have been shown to be effective in treating CRSwNP, though their mechanisms and long-term outcomes differ significantly. ESS is a surgical intervention designed to remove nasal polyps and restore normal sinus drainage, providing immediate relief by reducing polyp burden and improving airflow. In contrast, dupilumab works systemically by suppressing type 2 inflammation, leading to a slower but sustained reduction in polyp size and symptom relief. Studies comparing the two treatments highlight key differences in outcomes [45,46,47,48]. Patients undergoing ESS experienced a more immediate and significant decrease in nasal polyp burden in the short-term, compared to those treated with dupilumab. However, it must be noted that ESS is associated with a recurrence rate up to 40% within a few years after surgery. In contrast, dupilumab resulted in slower polyp reduction but gradual and long-lasting reductions in NPS and symptom burden [45,46,47,48]. One of the most compelling benefits of dupilumab is its superior impact on olfactory function, an area where ESS often falls short. In comparative studies, patients receiving dupilumab showed significantly greater improvement in smell function and overall quality of life [45,46,47]. Moreover, dupilumab led to significant higher relief in extranasal rhinologic symptoms such as cough and postnasal drainage [47]. However, dupilumab treatment requires continuous administration to maintain its effects, which raises considerations around long-term therapy costs and adherence. While ESS offers more immediate physical removal of nasal polyps, dupilumab provides systemic anti-inflammatory benefits, making it a more suitable option for patients with recurrent polyps, contraindications to surgery or those with comorbid conditions such as asthma [45,46,47,48].

A significant proportion of CRSwNP patients do not respond well to treatment with dupilumab [28,29]. This highlights the importance of identifying predictive biomarkers to assess how patients with CRSwNP will respond to dupilumab treatment. Several clinical and biological biomarkers have been studied as potential predictors of response to dupilumab therapy in CRSwNP, yet consistent evidence supporting their reliability remains lacking [30,31]. The ideal predictive biomarker should be readily available, reproducible, quantifiable and cost-effective, such as a blood parameter. Speaking of, very recently, Brkic et al. [39] were the first to report results of blood-based systemic inflammation indices, namely NLR and PLR, as predictors of response to dupilumab therapy in CRSwNP. The authors found that higher levels of pretreatment PLR were associated with a higher probability of reaching a NPS of 0, while NLR did not show this association. However, it must be considered that patients with residual nasal polyps can also show an excellent response to dupilumab therapy, without reaching a NPS of 0. Therefore, we included the recommended objective and patient-reported outcome measure into our study response criterion. In contrast to Brkic et al. [39], we were unable to identify a significant association between PLR, NLR, and dupilumab treatment outcome, neither in the group comparison, nor in the correlation and regression analysis. Another recent study by Zinellu et al. [40] found a similar positive association between elevated PLR and response to dupilumab treatment in patients with AD, another type 2 inflammatory disease. Moreover, the dupilumab response was also linked to higher levels of SIRI, SII, and AISI, further CBC-derived systemic inflammatory indices. To date, no study has investigated the prognostic ability of these biomarkers on the response to dupilumab therapy in patients with CRSwNP. Therefore, we aimed to test this hypothesis in the current study through performing responder-groups comparisons, correlations, and logistic regression analyses. Unfortunately, we failed to find a significant association between SIRI, SII, AISI, and dupilumab treatment outcome. Although, our negative results support the fact that the underlying mechanism of a possible association between CBC-based systematic inflammatory biomarkers and response to dupilumab treatment in type 2 diseases remains elusive.

We observed a significant positive correlation between pretreatment blood serum EOS levels and the total reduction in NPS, suggesting that higher pretreatment EOS values are associated with greater reductions in NPS. In contrast, no significant correlation was found between pretreatment EOS levels and the total reduction in SNOT-22 scores. In terms of combined parameters into a single response criterion, no statistically significant difference in pretreatment EOS levels was observed between responders and non-responders. Furthermore, pretreatment EOS did not emerge as a significant predictor of treatment response in the binary logistic regression analyses. The literature on pretreatment serum EOS as predictor of dupilumab response in CRSwNP remains uncertain. A recent study observed higher SNOT-22 reductions in cases of elevated pretreatment EOS [49]. Similar results were reported by Ryser et al. [50]. In contrast, Fujieda et al. [51] summarized the two dupilumab phase 3 studies and found no significant differences in treatment outcome in CRSwNP eosinophil subgroups (mild vs. moderate vs. severe). Future prospective high-quality studies are warranted to evaluate the prognostic role of pretreatment EOS in response of dupilumab treatment.

Several potential limitations of this study should be mentioned. First, the relatively small sample size and the fact that the study was conducted at a single center may affect the ability to generalize our results to a larger population. Second, the retrospective and observational design of the study may introduce selection bias and result in unaccounted-for confounding factors. Third, we utilized only two out of the five EPOS/EUFOREA response criteria (NPS reduction of >1 and SNOT-22 score), as the remaining criteria (improved sense of smell test in objective smell test, reduced impact of comorbidities such as asthma, and no need of systemic COS/salvage ESS) were not routinely measured in our clinical setting [44]. However, it is important to note that the response group demonstrated significantly greater reductions in NPS, SNOT-22, and impaired sense of smell subitem compared to the non-response group. These findings still support the value of our data in assessing the efficacy of dupilumab in our patient population. Nevertheless, while the data we present is valuable, it would have been more robust if all five criteria were applied. Fourth, the absence of routine smell testing required us to rely on patient-reported outcomes for impaired sense of smell as indication criterion, which lack the precision of objective measures and is not supported by guidelines [1,44]. Additionally, the six-month follow-up period may not be sufficient to assess the long-term impact of dupilumab treatment. It is also worth noticing that many patients continue their follow-up visits in outpatient settings, which restricted our capacity to collect long-term data beyond the initial six months. Nevertheless, the majority of CRSwNP patients receiving dupilumab experience more than an 80% reduction in NPS and symptoms within the first 8–12 weeks [44]. Future prospective multicentric studies with standardized follow-up and comprehensive data collection are needed to confirm these findings and identify reliable predictors of response to dupilumab in CRSwNP.

## 5. Conclusions

This study findings indicated that pretreatment EOS levels may be associated with nasal polyp reduction in patients with CRSwNP treated with dupilumab. Other CBC-inflammatory biomarkers did not show significant predictive value. Future research is needed to validate these findings and explore reliable biomarkers for predicting response to dupilumab treatment in CRSwNP.

## Figures and Tables

**Table 1:** Demographic and clinical characteristics stratified by response groups.

Characteristic	Responders (n = 58)	Non-Responders (n = 22)	*p*-Value
Sex, w/m	27/31	9/13	0.802
Age at start of treatment	51.9 ± 14.6	47.8 ± 14.2	0.256
Previous ESS	2 (0–7)	2 (1–7)	0.422
n° (%) of indication criteria, 3/4/5	12 (21%)/27 (46%)/29 (33%)	3 (14%)/9 (41%)/10 (45%)	0.547
Evidence of type 2 inflammation	55 (95%)	21 (95%)	0.909
Need of ≥2 courses systemic COS per year	42 (72%)	19 (86%)	0.247
Impaired sense of smell	56 (96%)	20 (91%)	0.571
Sense of smell in SNOT-22 subitem	3.9 ± 1.5	4 ± 1.1	0.925
Initial NPS	5 ± 1.6	4.3 ± 1.9	0.140
Initial SNOT-22	52.6 ± 21.8	63.2 ± 16.7	0.052
Coexisting asthma	40 (69%)	14 (64%)	0.790
Coexisting N-ERD	10 (17%)	5 (22%)	0.749
Leukocytes (×10^9^/L)	7.1 ± 1.5	7.2 ± 1.8	0.872
Platelets (×10^9^/L)	261.8 ± 56	254 ± 46.8	0.572
Neutrophils (×10^9^/L)	4.1 ± 1.2	4.4 ± 1.5	0.391
Monocytes (×10^9^/L)	0.6 ± 0.7	0.5 ± 0.2	0.540
Lymphocytes (×10^9^/L)	1.9 ± 0.5	1.8 ± 0.4	0.664
Total NPS reduction	4.2 ± 1.7	2.1 ± 2.2	<0.001
Total SNOT-22 reduction	36 ± 21.6	15.8 ± 14.8	<0.001
Total change in sense of smell SNOT-22 subitem	2.7 ± 1.7	1.1 ± 1.3	<0.001

Continuous parameters are presented as mean ± standard deviation. Categorical parameters are presented as absolute numbers and percentages (%). w: women. m: men. ESS: endoscopic sinus surgery. COS: corticosteroids. N-ERD: NSAID-exacerbated respiratory disease. SNOT-22: Sinunasal outcome test 22.

**Table 2:** CBC-based inflammatory biomarker data stratified by response groups.

Parameter	Responders (n = 58)	Non-Responders (n = 22)	*p*-Value
Eosinophils (×10^9^/L)	0.6 ± 1	0.4 ± 0.3	0.294
IgE (kU/L)	181.3 ± 191	246.8 ± 322	0.267
NLR	2.2 ± 0.9	2.5 ± 1.1	0.360
PLR	142.1 ± 40.1	139.4 ± 28.3	0.773
LMR	3.4 ± 1.2	3.5 ± 1.1	0.762
SIRI	1.5 ± 1.7	1.5 ± 0.9	0.917
SII	588.8 ± 275	633.4 ± 314	0.536
AISI	412.2 ± 533	386.5 ± 277	0.830

Continuous parameters are presented as mean ± standard deviation.

**Table 3:** Correlation analysis between total change in NPS and pretreatment CBC-based inflammatory markers.

Total Change in NPS	Eosinophils	IgE	NLR	LMR
Pearson’s ρ	0.248	0.086	0.013	0.103
*p*-value	0.027	0.447	0.912	0.361
**Total change in NPS**	**PLR**	**SII**	**SIRI**	**AISI**
Pearson’s ρ	0.117	0.016	0.144	0.152
*p*-value	0.300	0.886	0.202	0.178

**Table 4:** Correlation analysis between total change in SNOT-22 and pretreatment CBC-based inflammatory markers.

Total Change in SNOT-22	Eosinophils	IgE	NLR	LMR
Pearson’s ρ	0.168	0.086	0.055	0.043
*p*-value	0.135	0.447	0.629	0.703
**Total change in SNOT-22**	**PLR**	**SII**	**SIRI**	**AISI**
Pearson’s ρ	0.044	0.062	0.080	0.099
*p*-value	0.698	0.584	0.478	0.384

**Table 5:** Binary logistic regression analysis on treatment response.

Prognostic Factor for Treatment Response	Univariate Analysis			Multivariate Analysis		
	Odds Ratio	95% CI	*p*-Value	Odds Ratio	95% CI	*p*-Value
Age	1.02	0.9–1.1	0.253	1.03	0.9–1.1	0.215
Sex	0.79	0.3–2.1	0.651	0.73	0.2–2.6	0.626
Previous ESS	0.85	0.6–1.1	0.309	0.811	0.5–1.2	0.259
Asthma	1.27	0.4–3.5	0.650	1.22	0.4–4.1	0.736
NERD	0.71	0.2–2.3	0.576	0.65	0.2–2.6	0.546
Eosinophils	1.58	0.4–6.4	0.525	2.23	0.3–17.5	0.445
IgE	0.99	0.9–1.0	0.283	0.99	0.9–1.1	0.154
NLR	0.80	0.5–1.3	0.358	0.16	0.1–7.8	0.362
PLR	1.01	0.9–1.1	0.770	1.02	0.9–1.0	0.263
LMR	0.93	0.6–1.4	0.759	0.90	0.4–1.9	0.795
SIRI	1.02	0.7–1.4	0.916	11.37	0.1–96.5	0.480
SII	0.99	0.9–1.0	0.532	1.0	0.9–1.1	0.653
AISI	1.00	0.9–1.1	0.829	0.9	0.9–1.0	0.506

## Data Availability

The original contributions presented in the study are included in the article, further inquiries can be directed to the corresponding author.
